# Supplementary material for: Beyond medical prescription: Unveiling coping strategies’ role between perceived quality of care and treatment adherence among hypertensive patients
Source: PLOS Glob Public Health. 2025 Oct 15;5(10):e0004627. doi: 10.1371/journal.pgph.0004627 (PMC12527128; doi:10.1371/journal.pgph.0004627)
Supplement: S1 Text — This file includes variance inflation factors (VIF), tolerance values, and condition indices for all independent variables used in the regression models. (DOCX) [file pgph.0004627.s001.docx]

**SUPPLEMENTARY**

| **Coefficients^a^** | | | | | | | | |
| --- | --- | --- | --- | --- | --- | --- | --- | --- |
| Model | | Unstandardized Coefficients | | Standardized Coefficients | t | Sig. | Collinearity Statistics | |
|  |  | B | Std. Error | Beta |  |  | Tolerance | VIF |
| 1 | (Constant) | .967 | .098 |  | 9.886 | <.001 |  |  |
|  | Sex | .067 | .036 | .075 | 1.865 | .063 | .995 | 1.005 |
|  | Age | .002 | .001 | .070 | 1.754 | .080 | .995 | 1.005 |
| 2 | (Constant) | .639 | .130 |  | 4.916 | <.001 |  |  |
|  | Sex | .006 | .033 | .006 | .166 | .868 | .939 | 1.065 |
|  | Age | -.001 | .001 | -.027 | -.727 | .467 | .919 | 1.088 |
|  | Perception of Quality | .092 | .029 | .119 | 3.202 | .001 | .947 | 1.056 |
|  | Avoidance | -.015 | .027 | -.021 | -.557 | .578 | .941 | 1.063 |
|  | Task | .332 | .035 | .367 | 9.422 | <.001 | .866 | 1.154 |
|  | Emotional | -.014 | .022 | -.025 | -.667 | .505 | .950 | 1.053 |
|  | INTCEPQ | .005 | .040 | .005 | .122 | .903 | .953 | 1.050 |
|  | INTCAPQ | -.064 | .053 | -.045 | -1.211 | .226 | .964 | 1.037 |
|  | INTCTPQ | .268 | .059 | .169 | 4.510 | <.001 | .934 | 1.070 |
| a. Dependent Variable: adherence 19b | | | | | | | | |
