# Supplementary material for: Beyond medical prescription: Unveiling coping strategies’ role between perceived quality of care and treatment adherence among hypertensive patients
Source: PLOS Glob Public Health. 2025 Oct 15;5(10):e0004627. doi: 10.1371/journal.pgph.0004627 (PMC12527128; doi:10.1371/journal.pgph.0004627)
Supplement: S2 Text — This file presents the variance decomposition proportions used to assess multicollinearity among the predictors included in the final models. (DOCX) [file pgph.0004627.s002.docx]

**SUPPLEMENTARY**

| **Collinearity Diagnostics^a^** | | | | | | | | | | | | | | |
| --- | --- | --- | --- | --- | --- | --- | --- | --- | --- | --- | --- | --- | --- | --- |
| Model | Dimension | Eigenvalue | Condition Index | Variance Proportions | | | | | | | | | | |
|  |  |  |  | (Constant) | Sex | Age | Perception of Quality | Avoidance | Task | Emotional | INTCEPQ | INTCAPQ | INTCTPQ |  |
| 1 | 1 | 2.927 | 1.000 | .00 | .01 | .00 |  |  |  |  |  |  |  |  |
|  | 2 | .054 | 7.341 | .02 | .79 | .26 |  |  |  |  |  |  |  |  |
|  | 3 | .018 | 12.646 | .98 | .20 | .73 |  |  |  |  |  |  |  |  |
| 2 | 1 | 6.680 | 1.000 | .00 | .00 | .00 | .00 | .00 | .00 | .00 | .00 | .00 | .00 |  |
|  | 2 | 1.143 | 2.417 | .00 | .00 | .00 | .00 | .00 | .00 | .00 | .27 | .07 | .46 |  |
|  | 3 | 1.063 | 2.506 | .00 | .00 | .00 | .00 | .00 | .00 | .00 | .26 | .63 | .01 |  |
|  | 4 | .741 | 3.002 | .00 | .00 | .00 | .00 | .00 | .00 | .00 | .46 | .29 | .51 |  |
|  | 5 | .098 | 8.274 | .00 | .08 | .02 | .79 | .03 | .01 | .01 | .01 | .00 | .00 |  |
|  | 6 | .093 | 8.491 | .00 | .11 | .01 | .02 | .36 | .14 | .10 | .00 | .00 | .00 |  |
|  | 7 | .074 | 9.522 | .00 | .08 | .00 | .11 | .14 | .07 | .64 | .00 | .00 | .00 |  |
|  | 8 | .060 | 10.541 | .00 | .47 | .05 | .00 | .05 | .33 | .20 | .00 | .01 | .00 |  |
|  | 9 | .037 | 13.369 | .00 | .05 | .65 | .00 | .13 | .42 | .00 | .00 | .00 | .00 |  |
|  | 10 | .011 | 24.685 | .99 | .20 | .27 | .08 | .29 | .03 | .04 | .00 | .01 | .01 |  |
| a. Dependent Variable: adherence 19b | | | | | | | | | | | | | | |
