# Supplementary material for: Beyond medical prescription: Unveiling coping strategies’ role between perceived quality of care and treatment adherence among hypertensive patients
Source: PLOS Glob Public Health. 2025 Oct 15;5(10):e0004627. doi: 10.1371/journal.pgph.0004627 (PMC12527128; doi:10.1371/journal.pgph.0004627)
Supplement: Attachment — Submitted filename: Response Letter 1_Plos One Global Health.docx [file pgph.0004627.s004.docx]

# Response to Reviewers' Comments -

| Comment | Response | Location in Manuscript |
| --- | --- | --- |
| Reviewer #3 | | |
| The introduction is concise and avoids unnecessary details. However, the authors should kindly clarify what they meant **by 'high level of adherence' in the second sentence of the introduction** to enhance clarity of the sentence. | We appreciate the reviewer’s thoughtful comment. In response, we have clarified the phrase “high level of adherence” in the revised manuscript to specify that it refers to patients’ consistent adherence to treatment and their prescribed pharmacotherapy. This change has been made to improve clarity and enhance the reader’s understanding. | 1 |
| The authors should specify the **'themes**' referenced from 'Bhandari et al. [2]' in line 49, as the sentence lacks coherence. Specifying the themes will ensure clarity and context. | Thank you for this helpful suggestion. We have revised the sentence to specify the key themes reported by Bhandari et al. [2] for better clarity and coherence. These include misconceptions about hypertension and its treatment, behavioural modification difficulties (capability barriers), faith in alternative medicine and fear of treatment consequences (motivation barriers), as well as poor patient-provider communication, stigma, and socio-cultural influences (opportunity barriers). We also noted the perceived threat of the disease as a reflective motivator facilitating treatment adherence. These themes have now been clearly outlined in the revised manuscript. | 1 |
| The authors indicated in the manuscript that the prevalence of hypertension in Ghana is high, and hypertension care is typically generalised rather than tailored to individual needs. The authors should substantiate these assertions by providing appropriate **references** | We thank the reviewer for this valuable comment. We have now provided relevant references in the revised manuscript to support the statement regarding the high prevalence of hypertension in Ghana and the generalised approach to hypertension care. These references include recent national surveys and peer-reviewed studies that highlight both the burden of hypertension and the limited use of individualized treatment strategies in the Ghanaian healthcare context. | 6 |
| The authors should cite their source for the global NCDs target (reduction of HPT by 33% between 2010 and 2030) for easy reference. | We appreciate the reviewer’s observation. In response, we have cited the appropriate source for the global NCD target of a 33% reduction in the prevalence of hypertension between 2010 and 2030. The reference added is from the World Health Organization’s Global Action Plan for the Prevention and Control of Noncommunicable Diseases 2013–2020, which outlines this target as part of the global NCD agenda. The citation has been included in the revised manuscript for clarity and easy reference. | 4 |
| The authors should define **what they mean by '****coping strategies'** | The author has already defined what a coping strategy is. | 5 |
| The authors should re-evaluate the study design used. They mentioned using a retrospective observational study in the methodology section of the main body of the manuscript. However, their data collection procedure, capturing their response at a point in time, indicate that the study is more accurately characterised as a cross-sectional study rather than the cohort they claimed was adopted. |  |  |
| In the statistical analysis subsection of the manuscript, the authors indicated they describe the demographic characteristics of the study population by using measures of central tendency. The authors should kindly specify the measure of central tendency used in describing these demographic characteristics. Specifying the measures used will ensure appropriate interpretation of the data, including their distribution and nature, and will justify the choice of the statistical tests used in the study. | We have specified that we use mean. The rationale of bootstrap was indicated in the statistical section already | 9 |
| 9. The authors should consider specifying the statistical test used for the correlation analysis in the statistical analysis subsection and justify their choice with appropriate references, rather than stating just the visualisation tool (scatter plot). The scatter plot, moreover, depicted only the correlation between the gender differences in perception of quality of care and treatment adherence. | Spearman correlation and simple linear regression are indicated.  “The scatter plot, moreover, depicted only the correlation between the gender differences in perception of quality of care and treatment adherence.” That is exactly what the statistical section indicates. | 11 |
| The authors should consider describing the demographics with median instead of mean, as the mean may be misleading considering the assumed nature of the data (non-parametric assumption). | For this reason, we also reported additional measures of central tendency and dispersion (e.g., minimum, maximum, and mode) to provide a fuller picture of the data.  Regarding parametric assumptions, we respectfully clarify that such assumptions primarily apply to the **outcome variable** and model residuals in inferential analyses, rather than to descriptive summaries of predictor or background variables. Therefore, while reporting the median can be informative, using the mean in descriptive sections is not methodologically inappropriate, particularly when accompanied by other distributional indicators.  Besides we even use bootstrapping to address parametric assumptions | 12 |
| To enhance the understanding of the statistical tests used in the analysis, the authors should provide a clear definition or description of their variables, including the types of variables and their coding for the analysis. For example, regarding the 'comorbidities' variable, it should be specified whether it was treated as continuous or discrete, coded as categories a, b or c, represented in binary, or optioned yes or no, or quantified as the number of comorbidities, etc. | We have specified the level of measurement for all variables” Age, blood pressure (measured as the average of diastolic and systolic values), comorbidity, and admission rate were measured and treated as continuous variables.” | 9 |
| The authors should kindly justify the choice of variable inclusions in the regression models with reference. This justification should be incorporated in the methodology section | Variables or covariates were included in the models based on evidence from the literature indicating their influence on the outcome of interest. | 9 |
| The authors indicated in the limitations section that they employed a cross sectional design. However, in the methodology section they stated that they utilised a retrospective observational cohort design. The authors again indicated in the abstract that they used a prospective observational cohort study contrary to what they indicated in the main manuscript. This discrepancy raises concerns about the validity of their findings and the methodology used. The authors should rectify this inconsistency to ensure transparency and maintain integrity of their study | The study used a retrospective observational cohort design. It was a mistake to have indicated at the limitation section and other section that it was otherwise. Therefore, the incorrect study designs have been removed from the study ensure its consistency as suggested. | 21 |
| The abstract should specify the statistical tests and tools used in the study. | This is not standard practice. We indicated the type of analyses used | 2 |
| . What the authors consider as a 'statistically significant p-value should be clearly defined, as they reported a p=0.065 as a significant correlation (BP and comorbidities). This will ensure comprehensive understanding of the findings. | We have addressed this by saying “BP demonstrated a weak, positive correlation with comorbidities (Spearman's rho = 0.074, p = 0.065). | 13 |
